# “All of us are capable, and all of us can be scientists.” The impact of Scientist Spotlight assignments with undergraduates in physiology courses

**DOI:** 10.1152/advan.00116.2024

**Published:** 2024-08-08

**Authors:** Dax Ovid, Ashley Rose Acosta-Parra, Arsema Alemayehu, Jacob Francisco Gomez, Dathan Tran, Brie Tripp

**Affiliations:** ^1^Department of Physiology and Pharmacology, University of Georgia, Athens, Georgia, United States; ^2^Department of Neurobiology, Physiology, and Behavior, University of California-Davis, Davis, California, United States

**Keywords:** curriculum, inclusion, representation, scientist stereotypes, undergraduate

## Abstract

To advance ongoing efforts to diversify the healthcare field and promote inclusion in physiology education, the present study investigates the potential for an evidence-based intervention, Scientist Spotlight assignments, to highlight counterstereotypical representations of scientists in the context of majors and nonmajors physiology courses. Undergraduate students at an emerging Hispanic serving R1 institution completed six Scientist Spotlights assignments in their physiology courses. We conducted semistructured interviews and disseminated an established pre- and postsurvey protocol at the beginning and end of the courses. Our findings from interviews with 31 students from a range of marginalized backgrounds revealed that *1*) the biographical information about counterstereotypical scientists deeply resonated with students by humanizing science, *2*) the instructor’s implementation of the assignments made a meaningful difference in their feelings of inclusion, and *3*) the assignments supported students’ beliefs about their content learning and understanding of physiological concepts. The results from the survey showed that regardless of being in a major (*n* = 159) or nonmajor (*n* = 117) course, students from a range of demographic groups can and do significantly shift in their relatability to and descriptions of scientists. We highlight implications for inclusive curricula like Scientist Spotlights for addressing the issue of representation in physiology textbooks, curriculum, and healthcare fields at large.

**NEW & NOTEWORTHY** Scientist Spotlights significantly enhance undergraduate students’ feelings of inclusion and learning in undergraduate physiology courses at an emerging Hispanic-serving institution. By engaging with assignments featuring counterstereotypical scientists, students in both majors and nonmajors physiology courses shifted in their relatability to and descriptions of scientists. These results suggest that an inclusive curriculum, combined with supportive instructor practices, can positively impact student success and representation in physiology education.

## INTRODUCTION

There is a widespread push for designing and integrating inclusive curricula in science, technology, engineering, and mathematics (STEM) courses to address the inequitable outcomes for people from marginalized backgrounds in STEM (1, 2). The consensus study on *Advancing Anti-Racism, Diversity, Equity, and Inclusion in STEMM Organizations* lists several formal recommendations from National Academies of Sciences in Engineering and Medicine (NASEM) (3) on changes to be made to curriculum, including, “…adapt curriculum, physical environment, media stories, and other content to incorporate more examples of minoritized role models” (p. 11). This is particularly salient in prerequisite courses that lead to professional healthcare programs. Disparities in medical care have been linked to a lack of racial concordance between patients and healthcare providers (4–6), beckoning the need to increase racial and ethnic representation in the medical field. Thus evidence-based interventions for these prerequisite courses remain critical to advancing efforts to foster inclusion and increase diversity in medical healthcare professionals (7). To meet this charge, undergraduate students from marginalized backgrounds should engage with curricular materials that feature scientific role models that share similar characteristics to themselves. Previous scholarship with interventions that teach students about counterstereotypical scientists has shown the positive impact such assignments have on increasing aspects of students’ science identity and relatability to scientists, decreasing students’ stereotypical descriptions of scientists, and even increasing students’ academic performance (8, 9). Thus featuring counterstereotypical scientists in undergraduate physiology courses is an evidence-based strategy to meet the recommendations set forth by NASEM.

### Theoretical Frameworks of Scientist Spotlights Scholarship

The concept of scientist stereotypes and the theory of possible selves have influenced the design of Scientist Spotlights (10) including this study. Below, we briefly review these conceptual and theoretical frameworks and highlight how they informed the current study on undergraduate science students’ perceptions of scientists and themselves in relation to science.

First, *scientist stereotypes* continue to persist, namely, the stereotype of white, nondisabled, cis-gendered, and older men in laboratory coats (11). Despite these long-standing scientist stereotypes from countless studies in education research, the shifting representation of scientists in biology textbooks has been slow to respond with counterstereotypical representation (12, 13). Unfortunately, even medical and physiological textbook images continue to fail to represent our global population with regard to the diversity of skin tone, age, body size, ethnic background, disability, and LGBTQIA+ individuals (14–18). If we aspire to have culturally responsive physiologists, educators, and health care practitioners, then physiology instructors should intentionally curate our curriculum to ensure the representation of counterstereotypical scientists and medical images, which may include using assignments like Scientist Spotlights.

Second, *possible selves theory* considers what shapes an individual’s sense of “what they might become, what they would like to become, and what they are afraid of becoming” (19). Education research provides several interventions designed to expand the possible selves of middle and high school students (20–24). The possible selves theory is not about student assimilation into dominant cultural norms. Rather, this theory expands the definitional boundaries that students perceive for “scientists” to ultimately include “who they already are.” These definitional boundaries are shaped by the scientists that students encounter through their formal and informal educational experiences and their own communities and families.

Together, these theories underpin the design and assessments of Scientist Spotlights and guide the present study. To challenge long-standing scientist stereotypes and to push the definitional boundaries of scientists to include more diverse groups of students, we assessed this curricular intervention in the context of majors and nonmajors physiology courses. Below, we elaborate on the unique structure of Scientist Spotlight assignments.

### Scientist Spotlights

For nearly a decade, Scientist Spotlight assignments have emerged as a way to effectively increase positive outcomes for students from marginalized backgrounds. These assignments, accessible at https://www.scientistspotlights.org, teach course content through the narratives of counterstereotypical scientists, such as those who are disabled, from low-income or working-class backgrounds, racially or ethnically marginalized, and/or identify as LGBTQIA+. In these assignments, students read a summary about a scientist alongside their photo, explore links related to the scientist’s biography and research, and then write a metacognitive response to reflection questions, including, “What do these resources tell you about the types of people that do science?”

Developed from the educational research of a community college professor (8, 10), Scientist Spotlight assignments have led to significant changes in several areas. After completing these assignments, college students’ descriptions of scientists shifted from stereotypical (white, male, elite) to more inclusive (all types of people). Additionally, students reported feeling a greater connection to scientists. Those who engaged with the Scientist Spotlight assignments achieved, on average, a course grade level higher than a comparison group that learned about the scientists without engaging in written reflections (8). Many other studies have since explored the impact of these assignments. Completion of Scientist Spotlight assignments showed significant shifts in students’ descriptions about and relatability to scientists across a range of age groups and contexts at other community colleges (25, 26); in a masters-granting, minority-serving institution’s biology department (27); in courses at R1 institutions (28–30); and even at the high school level (9).

One avenue that has yet to be explored is which aspects of these assignments cause students to increase their relatability to scientists: the biography, reflection questions, scientist’s research, or something entirely different? Additionally, there is little evidence on how to implement Scientist Spotlights in a classroom to yield the best results. Although the vague details of how to implement such a curriculum may allow for context-relevant adaptability, it also may sacrifice key elements that make a meaningful difference for STEM students who experience an inclusive STEM curricular supplement. Elucidating how the fidelity of implementation (31) or the ways in which instructors implement these assignments impacts student outcomes is essential to create a practitioner’s guide on best implementation practices.

To characterize if and how an inclusive STEM curricular supplement resonates with undergraduate students from marginalized backgrounds, we implemented Scientist Spotlight assignments in two physiology courses and conducted semistructured interviews to explore how undergraduate students perceived these assignments and what aspects were most meaningful to them.

## METHODS

### Authors’ Positionalities

Our team consists of individuals from a variety of backgrounds, each bringing unique perspectives and experiences that enrich our collective understanding of this project. The authors represent marginalized groups, including People of Color, LGBTQIA+ individuals, those with learning disabilities, first-generation college students, and people from low-income backgrounds, as well as those from more privileged identities, such as white, presently nondisabled, and highly credentialed individuals. We are aware of the power dynamics inherent in these identities and recognize the privileges and disadvantages they entail in various contexts. The authors’ lived experiences with societal oppression and discrimination have shaped every aspect of this project.

### Internal Review Board Approval

This project was approved by the University of California, Davis’s Internal Review Board under exempt status (Protocol No. 1825759-2).

### Study Context

Our study was conducted at an emerging Hispanic-serving R1 university on the West Coast in two human physiology courses: a lower-division nonmajors’ course and a majors upper division course. A group of undergraduate researchers unrelated to the courses selected several Scientist Spotlight assignments from the online database (www.scientistspotlights.org) that aligned with their interests and complemented physiology topics covered throughout the courses. The senior author taught both courses and implemented the same six Scientist Spotlight assignments in each course (see Supplemental Material C; all Supplemental material is available at https://doi.org/10.6084/m9.figshare.26305123). We chose six of these assignments based on literature that identified four or more Spotlight assignments that had the greatest outcomes for students (30). The findings reported here are part of a larger study about how inclusive curriculum aligns with and can better support cultural learning pathways for undergraduate students.

### Implementation

The six Scientist Spotlight assignments were implemented in the same manner across both physiology courses by the same instructor (senior author) during the Spring quarter of 2022. The assignments were offered in weeks 4–9 of the quarter based on the course content that aligned with the scientists’ research. For example, the Spotlight assignment on Dr. Agnes Day who is a cancer researcher was assigned in week 4 in which immunology content was covered.

The instructor introduced the assignments in class at the beginning of each week they were assigned by showing one slide that contained a picture of the scientist with a short blurb about their research and how it related to the course content. Of importance, she never highlighted their counterstereotypical identities to avoid tokenization. In the following week, the instructor selected two student responses that were thoughtful and met all grading criteria to anonymously share with the class and collected students’ consent before doing so.

Students had 7 days to complete the assignment. The assignments were embedded in the Learning Management System Canvas and were worth 25% of the students’ total grade (∼4% for each assignment). They were not graded for accuracy; instead, the assignments had a minimum sentence criterion of at least five sentences. Students were graded on whether they addressed and reflected on both parts of the assignment: the biographical source and the scientists’ research. Students were invited to answer any of the following four questions in their response: what was most interesting to you in reviewing these resources; what did you learn from these resources about [insert scientists’ research topic]; what new questions do you have after reviewing these resources; and what do these resources tell you about the types of people who do science?

### Student Interviews

Using a mixed-methods approach, we distributed a pre- and postsurvey before and after the Scientist Spotlight assignments to collect student perceptions of these assignments from the two courses through an online survey (*n* = 357; see details below for quantitative methods). At the end of the survey, we asked students if they were interested in participating in an interview. Of the students who expressed interest in participating in interviews in the postsurvey (27%, *n* = 98 survey respondents), we invited a subset of students from a range of representative backgrounds to participate in semistructured interviews (see Supplemental Material A for interview questions).

The interview questions were piloted and revised to address the research questions and ensure face validity (32). We conducted and recorded the interviews on Zoom. The audio recordings were then transcribed and deidentified using pseudonyms to protect the anonymity of participants. Using deductive content analysis (33), three researchers independently read through 10% (*n* = 3) of the interviews, annotating salient ideas related to our research questions and expected findings from survey responses (described above). We then reconvened to discuss similarities of emerging ideas and constructed categories that described collective ideas. This created an initial codebook that we used to repeat this process with another 10% of interviews while identifying new categories that emerged and modifying our codebook to include these categories. We repeated this process three times until data saturation was reached (i.e., no new categories emerged). Two of the three researchers then independently coded 10% (*n* = 3) of the remaining interviews and reconvened to reach an interrater reliability (IRR) of greater than 80% which was established using MAXQDA 2020. Using the final codebook, all interviews were then recoded independently by one coder (half by one researcher and half by the other) in the MAXQDA software.

### Survey Design and Analysis

The survey design was adapted from previous studies in Scientist Spotlight Interventions (8–10) and consisted of the following:

- Qualitative prompts to assess *Relatability* (“I know of one or more important scientist(s) to whom I can personally relate.”) and *Stereotypes* (“Based on what you know now, describe the types of people that do science. If possible, refer to specific scientists and what they tell you about the types of people that do science.”);
- Quantitative closed-ended items, which consisted of 3 controls and 13 items to measure performance/competence (5 items), interest (2 items), and recognition (6 items), referred to as the *PCIR instrument* (adapted from Ref. 34; see Supplemental Table S1). Informed by qualitative study in science identity formation (35), the performance/competence, interest, and recognition (PCIR) is a closed-ended Likert survey that has been used to measure shifts in constructs associated with student engagement and persistence in STEM majors (34); and
- *Demographic questions* and the possibility to sign up to participate in an interview (postassessment only).

The pre- and postassessments are provided in Supplemental Material B.

Students who met our inclusion criteria (i.e., granted consent and completed both pre- and postsurveys) were included in our statistical analyses. For our statistical comparisons for shifts in students’ agreement with the Relatability prompt (i.e., Strongly Disagree/Disagree shifting to Agree/Strongly Agree), we used McNemar’s χ^2^-test with continuity correction (36) and conservatively adjusted our level for significance based on the number of pairwise comparisons based on Bonferroni corrections (i.e., majors course/nonmajors courses and by students’ self-reported race and ethnicity, gender, and sexual identity). As done in previous studies (9), if the Relatability prompt showed significant shifts across our groups of interest (majors and nonmajors), then we planned to combine the data for subsequent analyses for the Stereotypes prompt and PCIR results. We deductively coded the written responses to the Stereotypes prompt using the existing codebooks for qualitative analysis (8, 9) and inductively considered whether this particular set of responses warranted new categories. Finally, before using the PCIR instrument for quantitative analysis of shifts in aspects of students’ science identity, we first examined the evidence for the validity of this instrument with a novel population of students. This process for evaluating instrument validity was conducted with exploratory and confirmatory factor analysis (EFA/CFA) (37).

## RESULTS

In this section, we present findings from the semistructured interviews and surveys conducted with undergraduate students who completed six Scientist Spotlight assignments in two physiology courses (a majors and nonmajors course).

### Interviews

All participants identified with one or more of the following marginalized identities: LGBTQIA+ (39%, *n* = 12), first-generation (52%, *n* = 16), and/or People of Color (87%, *n* = 27). Three salient themes emerged from our qualitative data analysis of 31 undergraduate student interviews. All students (100%, *n* = 31) responded to the interview question, “Which aspects of Scientist Spotlights resonated most with you?” From these responses, 81% (*n* = 25) of students described the scientists’ biographies, 45% (*n* = 14) identified the reflection questions, and 42% (*n* = 13) described the scientists’ research as impactful. In addition, two unexpected themes arose from the interviews in which 48% (*n* = 15) of students articulated the importance of how instructors implement Scientist Spotlight assignments and 30% (*n* = 8) explained how these assignments positively impacted their ability to learn course content by reducing memorization. There were no differences in these findings across the two courses, and thus we present these data in aggregate in the following subsections.

#### Resonation of inclusive curricula.

The Scientist Spotlight assignments consisted of three primary sections: the research of a counterstereotypical scientist, their biography, and a set of reflection questions for students to complete (see Supplemental Material D for examples). Seventy-seven percent (*n* = 24) of students identified the scientists’ biographies, 45% (*n* = 14) resonated with the reflection questions (*n* = 14), and 42% (*n* = 13) identified the scientists’ research as a part of the assignment that most resonated with them (Table 1). Of note, some students resonated with multiple sections of the assignments, thus, numbers within these subcategories do not add up to 100% of our population. Many students reflected on the ways in which the personal stories of scientists helped humanize science:

“I think the biggest aspect that resonated with me was the human aspect, and that’s something I didn’t ever get before in a science class. Bringing the human aspect to science, not just dropping the content on us, but saying, ‘Hey, these are real people who study and have improved the field of what you’re studying.’ And then also the flip side, who is the science for. It’s not just the people who study science, it’s also those who benefit from the science, which could be different disadvantaged communities…” (Bond)

Some students found the scientists’ backgrounds as a source of inspiration and hope for pushing through challenges:

“I like the background aspect the most, because it’s nice to see somebody from where they came from and what they have grown up to be. And that just inspires me more. You just feel like you can do more when you see that other people have gone through a lot harder challenges than you have, or similar challenges that you have gone through, and it just pushes you forward, gives you hope that you can do that too.” (Huda)

Almost half of students resonated with the reflection questions, highlighting how the questions encouraged them to think critically about the connections between the identities of scientists and who does science:

“Reflection questions resonated most because after finding out what they worked on and their experiments and stuff, it made me really think about who I was looking at because of those reflection questions. They would always ask, ‘What’s your view of people who do science?’ or ‘How do you feel about this person now after reading their work?’ And then I really had to dig deep and actually think about who they were as a person and what that meant for science. There were really good questions to reflect on, so I think that was the most impactful.” (Amira)

Finally, well over one-third of students identified the scientists’ research as impactful. For example, Andrew resonated with the research portion by describing a connection between the topic of research and cultural responsibility:

“There was one scientist who did research for heart disease in South Asian communities. I really like that because they found that people from that region were at more of a risk [for developing heart disease]. And if you never researched that specific group, or if there was no representation, the research would be the same for all sorts of people. But no, that’s not true. If you’re recommending a treatment for heart disease that only works for a different region of people from a different country or a different area, and that’s not accurate. That’s not the best healthcare you could give to this person. So that really resonated with me.” (Andrew)

#### Implementation matters.

Although teaching methods were not an initial consideration for our research questions, over half of students (55%, *n* = 17) in this study shared that instructor implementation affected their experience of Scientist Spotlights (Table 1). Students highlighted in their interview responses aspects of implementation which included *1*) how instructors shaped classroom culture overall, *2*) how instructors preframed the assignment, and *3*) how instructors evaluated and graded the assignment.

**Table 1.** Example quotes from undergraduate students regarding resonation and implementation and of Scientist Spotlight assignments

Themes (%, *n*)	Subcategories	Example Quotes
Resonating aspects of inclusive curricula (100%, *n* = 31) *Described which aspects of Scientist Spotlight assignments resonated with students*	Scientist biographies (81%, *n* = 25)	“I Think What Resonated Most with Me is That I Was Learning about People Who Find [Their] way to Science in [Their] Own Way and Having These Different Identities Really Resonated with Me, ‘Cause like I Said, I Think That I Found my Way to Science Not Because It Was Placed in Front of Me, but Because I Liked Books and I Liked Reading, and Then I Saw These Colorful Pictures and I Was like, Oh, Let Me Try This out, and so I Think Just Learning about the Journey and Learning That Different People Can Get to Science Different Ways, and I Think That Really Resonated with Me.” (Sabrina)
	Assignment reflection questions (45%, *n* = 14)	“I think [reflection questions] definitely had the most impact, just even the writing portion of it, because you just have to remember your thoughts, and write them down on a piece of paper. I kinda liked doing it just because I was able to break down what I thought about it and was able to kinda relate if I had certain experiences with what they were talking about, like if it happened in my family or in my life personally. [The questions] were able to help me dig deeper into why I relate; I mean, they were really good questions to help get started, but once you get past that helping jump point, I was able to just write what I was thinking about and how I was able to relate it to what I learned before or to how it can be applied to different scenarios or ask questions about it.” (Marisol)
	Scientists’ research (42%, *n* = 13)	“I feel the research behind it also really, really resonated with me ‘cause some of them talked about some diseases that run in my family or talked about something that I was considering maybe in the future, weight loss surgery, which when I read more about it, I was kind of scared but it definitely informed me about a lot of things.” (Jenifer)
Implementation matters (48%, *n* = 15) *Described how implementation of Scientist Spotlight assignments positively affected students*		"Reading the Scientist Spotlights, and also just being in a class with [the instructor], she made it really inclusive and she made me feel comfortable every time I was in class. So I think having a positive environment or like an environment where you’re welcomed, and individuals or teachers make it known like, ‘This is a safe space,’ it’s really beneficial.” (Violeta)
		“The professor really cared a lot about the class and the Scientist Spotlights, and that’s something that I think is very rare these days. A lot of professors they’ll just be like, “Oh, I gotta teach this class if I want to keep my research up,” or, “I need to teach only the course content relevant to this class topic. But in this class, [the instructors and teaching assistants] really cared about the students learning about different scientists and their backgrounds, which is something that I don’t really see that much nowadays in most classes.” (Greg)
		“I just think that learning about all these different people was very impactful, and I really liked how [the instructor] would tie in back to the lecture because it made it relevant to what we were learning. So I think that was most impactful because it was always brought back to our education and how this affects us…” (Cristina)
Improves content learning (30%, *n* = 8) *Described how Scientist Spotlight assignments enhanced student learning of content.*		“I like the scientist spotlight ‘cause there was more of an emphasis on diversity in science and then also application because you’re reading about these different scientists and their discoveries to what you’re learning about right then in class, so it’s more applicable.” (Alexa)
		“I feel I do relate to scientists more now. And then I was more interested in learning about that topic afterwards, after we did the scientists spotlight, I was like I actually enjoyed the [content] more when we were reading about it and learning about it in class.” (Cristina)
		“[The SSI assignments] definitely helped me more with comprehending course information. So, it worked out for me like it was supposed to.” (Jocelyn)

Students who alluded to the overall encouraging classroom learning environment spoke in generalities of the ways instructors advocated for inclusion in their everyday language throughout the course, for example:

“I definitely think that the professor was pretty insistent on the fact that all of us are capable and all of us can be scientists and then demonstrated that through the Spotlight assignments and giving us the opportunity to be successful in the class. I think I really appreciated that, and that made me feel like I could definitely be successful.” (Jocelyn)

One student even specifically remembered noncontent language that preframed the classroom activity:

“She presented this in a way like, ‘This is an opportunity to really open your mind up and just go into these assignments with an open mind and open heart and just learn what these scientists do.’ The professor I had was just great…I feel like if I had a different professor, my experience with the Scientist Spotlights might have been different.” (Reina)

Jenifer discussed how classroom conversations were encouraged and how Scientist Spotlight assignments assisted in increasing her relatability to counterstereotypical scientists and awareness of inequities:

“[The professor] allowed others to talk about their experiences, and she was very conscious about how some of these things can be controversial. She included articles like Scientist Spotlights to make us aware of inequities in how researchers are often presented, like white males, but I learned there are many researchers who don’t look like that. That really helped, because I am a Latina woman.” (Jenifer)

Some students described how the instructor followed-up after the the homework assignment, such as sharing anonymized student responses or making it directly relevant to course content:

“[The instructor] would present excerpts from what other people took away from the Scientist Spotlight. It was really like, ‘Wow, I thought about it this way, but other people think about the scientists this way…’” (Raspreet)

“That was the first time I think that I had taken a class that had these Scientist Spotlight assignments, or assignments that were worth that much of our grade, which I think really helped. Then the professor would relate them to what we were learning about in class, like the cardiovascular system or the different systems. During the PowerPoint, it was brought up, ‘Oh, we learned more about this content based on this scientist… We’re studying this, this scientist studied that. They researched this and it relates to our content because X, Y, Z.’” (Estrella)

#### Improves content learning.

Relating to when these assignments are implemented within the course structure, another unprompted category was how the inclusive curricula helped solidify course content for students (Table 1). Almost a third of students (29%, *n* = 9) reflected on the ways in which Scientist Spotlight assignments assisted them in making connections to and increasing lasting memory of course content. Students provided thoughtful reflections on how making connections between counterstereotypical scientists’ and the course content improved their sensemaking and ability to retain STEM information:

“Having to connect [the class content] back to a scientist, it was more interesting to see it play out. Like, they had a hand in discovering whatever this is, or they had research with whatever it was. It was interesting and easier to connect it back to class. It made making sense of things easier ‘cause you actually had to read this stuff and, yeah, make connections to what is being taught and really think.” (Raspreet)

“Relating to the scientist makes me, obviously, so much more interested in reading that research article. And then applying my [course content] knowledge to that, it kind of connects the dots for me. And so, I’d be more likely to retain that information as well.” (Esha)

“[The SSI assignments] definitely helped me more with comprehending course information. So, it worked out for me like it was supposed to.” (Jocelyn)

Many students, like Sadie and Jenifer, made references to how the research conducted by the countestereotypical scientists assisted them with remembering content for assessments:

“On an exam, I was like, these questions related to the research I’m remembering the most because I know this. This is not content I even had to worry about just because I knew their stories, and it was super cool.” (Sadie)

“So I feel like the Scientist Spotlights really bring out a different aspect and it also relates to the course, too. One of them was talking about cell disease and stuff and that was really helpful with course information. I mean, personally, I still remember the stuff too, so it definitely sticks.” (Jenifer)

Some students reflected on how Scientist Spotlights allowed them to connect science to social models in other courses:

“I like to do this thing where I kind of make sure that I can compare one thing from one class to another and kind of intertwine it, because then I feel like, “Okay, I’m getting my degree’s worth,” [chuckle] and I think this class really did that, especially in regards to how I learned about the medical model and the social model [in the Spotlight assignments] to some of the different terms we use in my Linguistics class fall quarter.” (Fawn)

### Surveys

In alignment with observations in prior studies (8, 9), we found statistically significant shifts in various aspects of students’ relatability to and descriptions of scientists. This serves as the first study to show these shifts for both majors (*n* = 159) and nonmajors (*n* = 117) taught by the same physiology instructor.

The self-reported demographics of majors who agreed to participate in the study include 157 students who disclosed their gender (4% trans/gender nonconforming, 72% cis-female/women, and 24% cis-male/men), 157 students who disclosed their race or ethnicity (82% students of color and 18% white), and 158 who disclosed their sexuality (16% LGBTQIA and 84% heterosexual/straight). Nonmajors who agreed to participate in the study included 106 students who disclosed their gender (4% trans/gender nonconforming, 83% cis-female/women, and 13% cis-male/men), 116 students who disclosed their race or ethnicity (84% students of color and 16% white), and 116 who disclosed their sexuality (25% LGBTQIA and 75% heterosexual/straight).

#### Students significantly shift in relatability to scientists.

**Figure 1.**
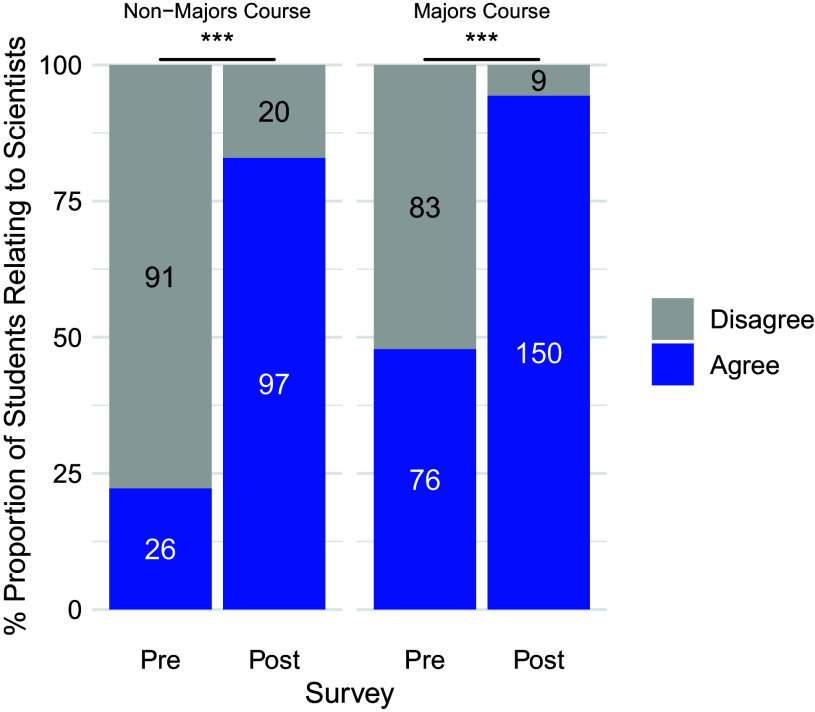
Significant overall shifts undergraduates’ relatability to scientists, before and after Scientist Spotlight assignments in a nonmajors and a majors physiology course. The proportion of students who Agree in blue (“strongly agree” and “somewhat agree”) and Disagree in gray (“strongly disagree” and “somewhat disagree”) with the Relatability prompt. McNemar’s χ^2^-tests show pre- and postdifferences are significant at ***P < 0.0001, with Bonferroni’s adjustment for significance set at *P* < 0.00313.

**Figure 2.**
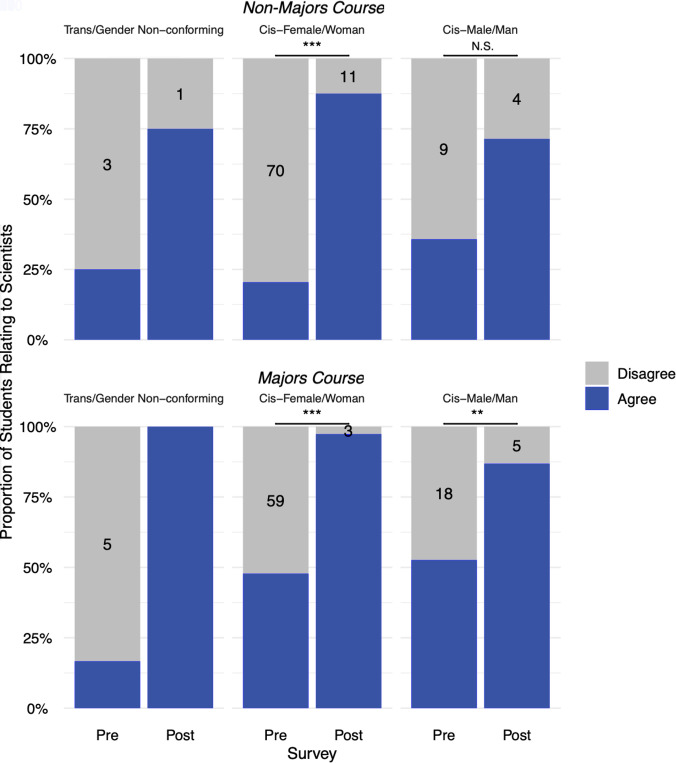
Relatability prompt agreement, disaggregated by self-reported gender: shifts undergraduates’ relatability to scientists, before and after Scientist Spotlight assignments a in nonmajors and a majors physiology course. The proportion of students who Agree (blue) and Disagree (gray) with the Relatability Prompt. McNemar’s χ^2^-tests show pre- and postdifferences are significant at **P < 0.001 (Majors, Cis-Male/Man) and ***P < 0.0001 (Majors and Non-Majors, Cis-Female/Woman). Pre- and postshifts for Non-Majors, Cis-Male/Man were not significant, with Bonferroni’s adjustment for significance set at *P* < 0.00313.

**Figure 3.**
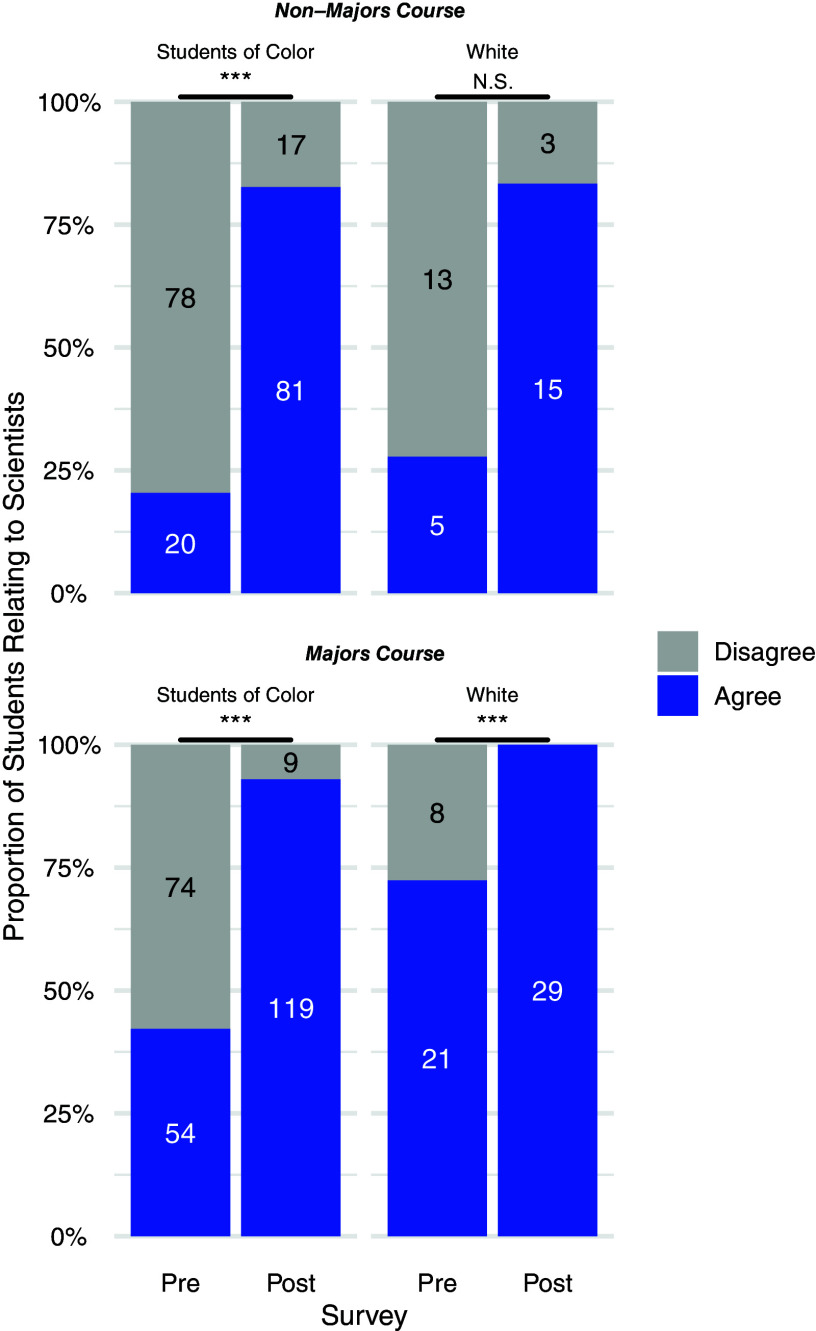
Relatability prompt agreement, disaggregated by self-reported race and ethnicity: shifts undergraduates’ relatability to scientists, before and after Scientist Spotlight assignments in a nonmajors and a majors physiology course. The proportion of students who Agree (blue) and Disagree (gray) with the Relatability Prompt. McNemar’s χ^2^-tests show pre- and postdifferences are significant at ***P < 0.0001 (Majors and NonMajors, Students of Color; Majors, White). pre- and postshifts for Non-Majors, White were not significant, with Bonferroni’s adjustment for significance set at *P* < 0.00313.

**Figure 4.**
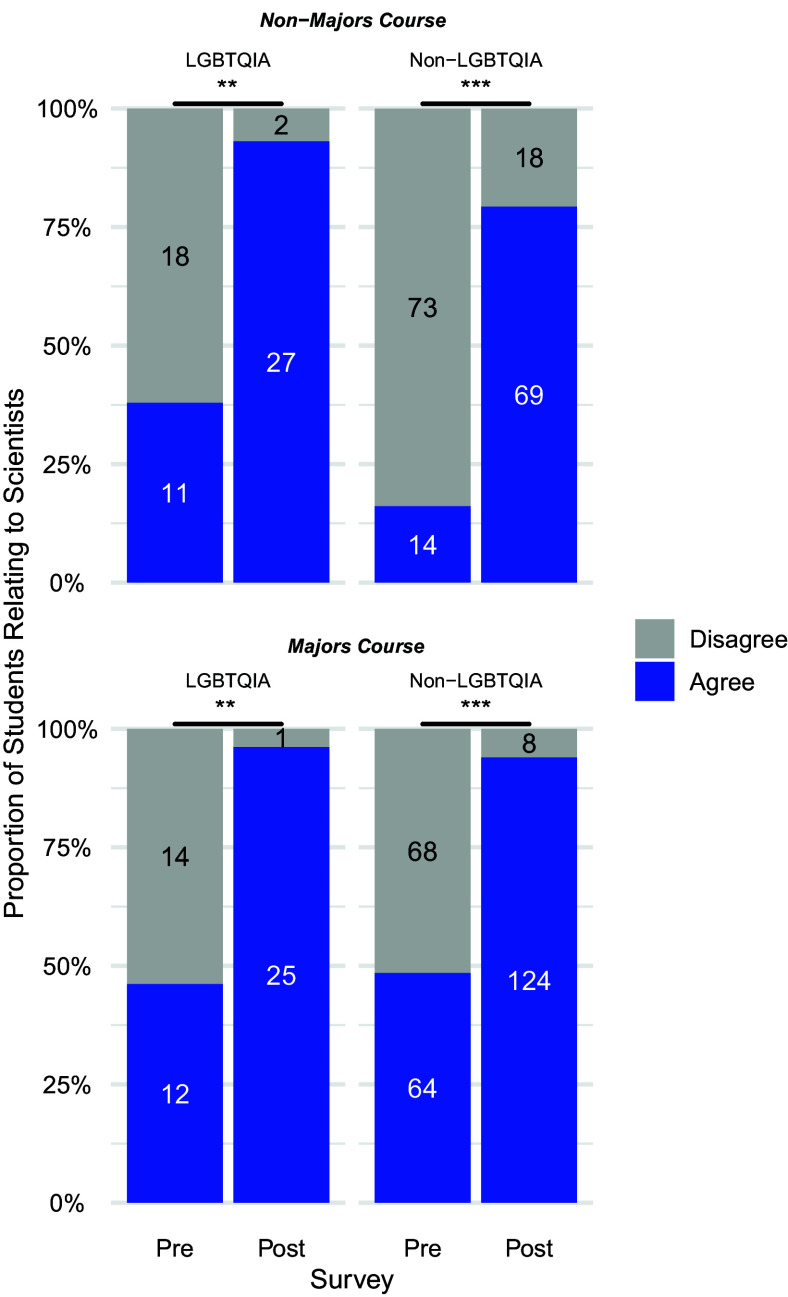
Relatability prompt agreement, disaggregated by self-reported sexuality: shifts undergraduates’ relatability to scientists, before and after Scientist Spotlight assignments in nonmajors and a majors physiology course. The proportion of students who Agree (blue) and Disagree (gray) with the Relatability Prompt. McNemar’s χ^2^-tests tests show pre- and postdifferences are significant at **P < 0.001 (Majors and Non-Majors, LGBTQIA) and ***P < 0.0001 (Majors and Non-Majors, Non-LGBTQIA), with Bonferroni’s adjustment for significance set at *p* < 0.00313.

For the Relatability prompt within both the majors and nonmajors course, pre- and postshifts in agreement were significant at the course level (Fig. 1) as well as disaggregated comparisons by gender (Fig. 2), race and ethnicity (Fig. 3), and sexuality (Fig. 4) for the students who self-disclosed. Through McNemar’s χ^2^-analysis, there were significant shifts for all comparisons (Bonferroni’s adjustment for significance set at 0.00313 for 16 comparisons), except for students in the nonmajor course who identified as White (*P* = 0.004) or as cis-men (*P* = 0.131); however, it is important to note the small *n*-value for these two groups. The number of nonmajor, cis-men students who could relate to one or more important scientists doubled from 5 in the pre to 10 in the post (out of 14 total), and the number of nonmajor, White students tripled from 5 to 15 (out of 18) who could relate. Supplemental Table S2 shows a summary of these 16 statistical comparisons for pre- and postshifts in agreement with the Relatability prompt. Table 2 highlights pre- and postwritten explanations that students provided to explain their reasoning.

**Table 2.** Examples of pre- and postresponses to Relatability Prompt: “I know of one or more important scientists to whom I can personally relate”

Pseudonym	Preassessment	Postassessment
Lirthika	“I don’t know if I can relate to any of them simply because I don’t know much about their personal life besides their contributions to their fields.” *(Relatability: “Strongly Disagree”)*	“I think Dr. Kiranbir Josan trying to point out problems in the South Asian population allowed me to relate to her. My family is Asian Indian and because of our lectures on hormones/insulin and Dr. Josan’s presentation I’ve found that many of my mom’s recent symptoms line up with type 2 diabetes. I’ve been taking care of her and soon we’re visiting the doctor to have her symptoms checked. Hopefully I’m wrong but if not, then the scientist spotlight has helped us seek help.” *(Relatability: “Somewhat Agree”)*
Carmen	“As a Latina, I don’t think I am very familiar with or have ever met other women who share my identities and who also practice science.” *(Relatability: “Somewhat Disagree”)*	“I relate to Dr. Veronica Ades, as a Latina and Hispanic woman who is also interested in research. Dr. Ades is a fierce feminist who works with women who have experienced gender-based violence, and I also consider myself passionate about topics like these.” *(Relatability: “Strongly Agree”)*
Ahmad	“I’m not sure who qualifies as an important scientist. For that reason I am not sure how I can personally relate to an important scientist or if I can relate to one at all.” *(Relatability: “I don’t know.”)*	“Coming from a low-income background myself, seeing scientists such as Dr. Agnes Day speaks to me and inspires me as it shows the diversity that exists in the scientific community.” *(Relatability: “Strongly Agree”)*
Kevin	“I’m not really exposed to the field of science and any scientists I know that share a similar background as I. I think it’s important to have other scientists that you relate to so that you can know that you can also do science.” *(Relatability: “Somewhat Disagree”)*	“I can relate to Dr. Lim because he is a fellow Asian in science who pursued science and discussed weight loss surgeries. As an Asian in science, it is nice seeing someone who identifies similarly to me in science.” *(Relatability: “Somewhat Agree”)*

#### Students significantly shift in scientist descriptions.

**Figure 5.**
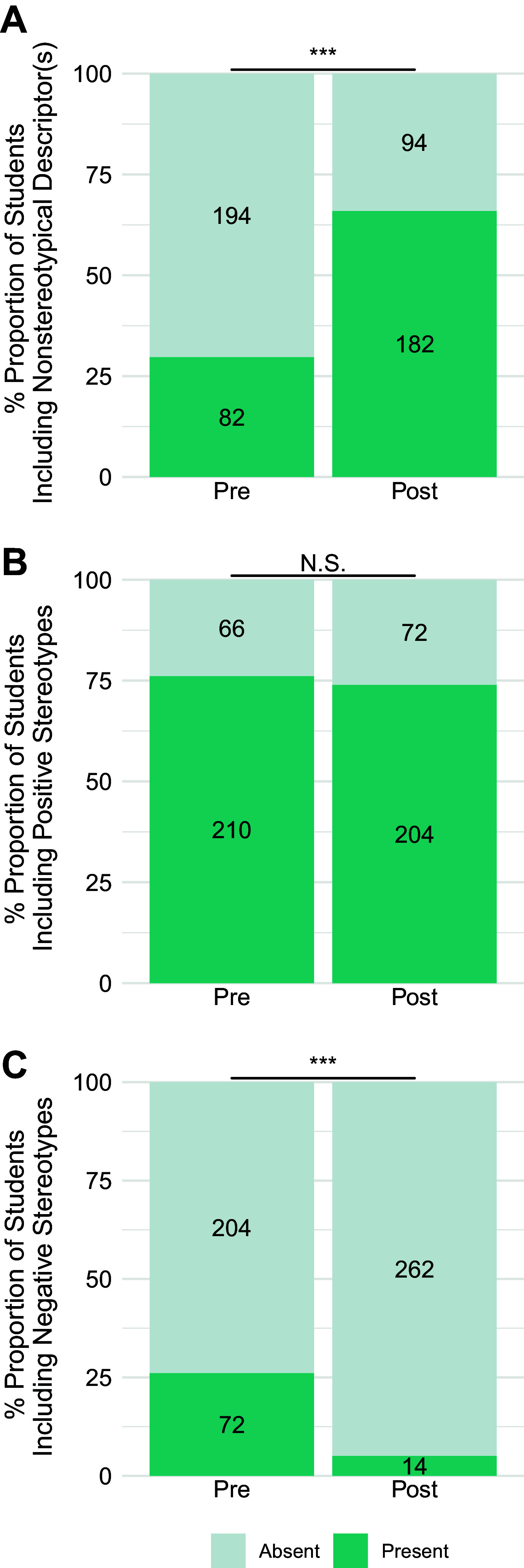
Stereotypes prompt descriptions, overall shifts in the proportion of undergraduates’ descriptions of the types of people that do science, before and after Scientist Spotlight assignments (combined nonmajors and a majors physiology courses). The proportion of students for whom Nonstereotypical Descriptor(s) (*A*), Positive Stereotypes (*B*), and Negative Stereotypes (*C*) were Present (teal) or Absent (gray). McNemar’s χ^2^-tests show pre- and postdifferences are significant at ***P < 0.0001 (increased Nonstereotypical Descriptor(s), decreased Negative Stereotypes). Pre- and postshifts for Positive Stereotypes were not significant (N.S.), with Bonferroni’s adjustment for significance set at *P* < 0.0125 for 3 comparisons.

**Figure 6.**
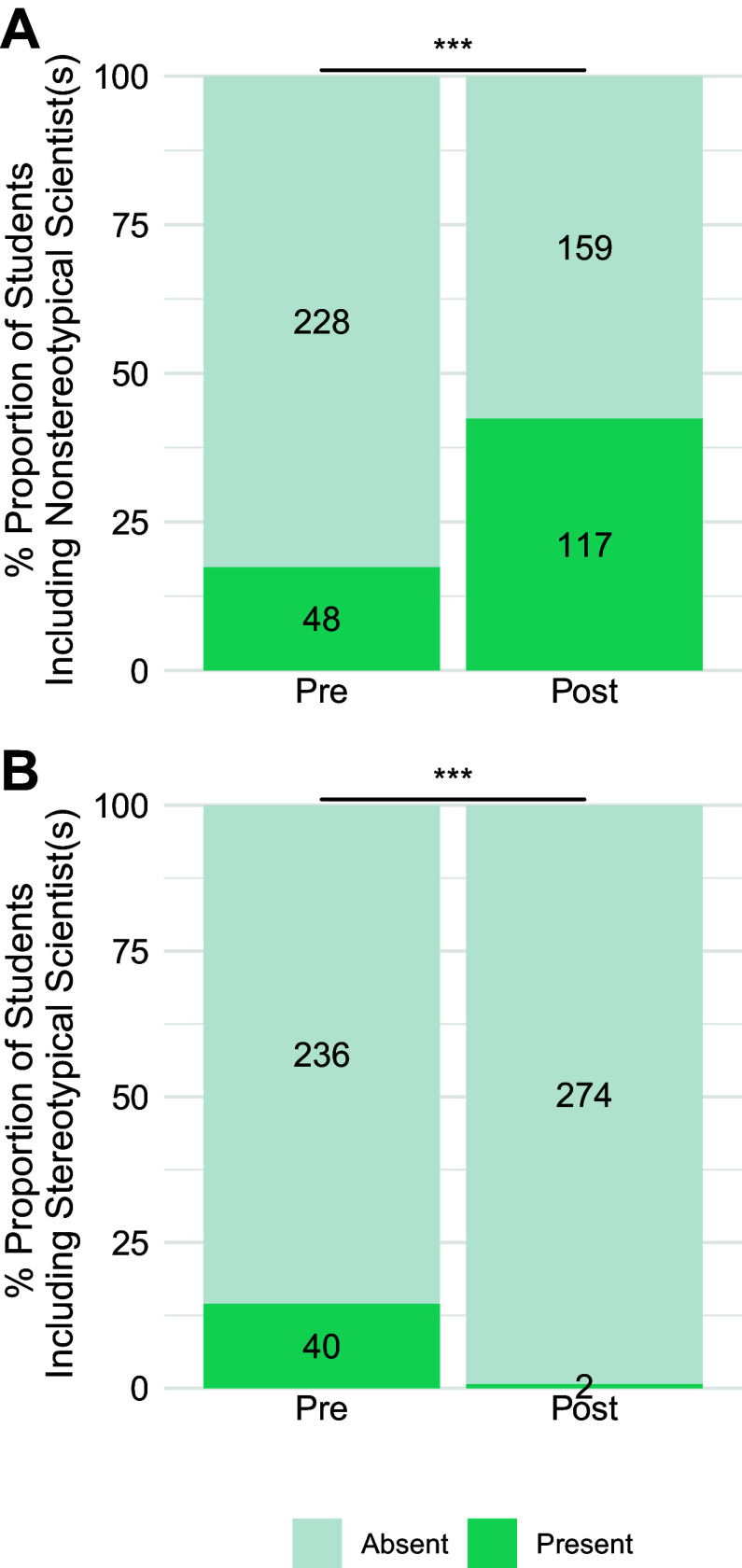
Stereotypes prompt descriptions, shifts in the proportion of undergraduates who included the names of Nonstereotypical (*A*) and Stereotypical scientists (*B*), before and after Scientist Spotlight assignments (combined nonmajors and a majors physiology courses). Names were Present (teal) or Absent (gray). McNemar’s χ^2^-tests show pre- and postdifferences are significant at ***P < 0.0001 (increased Nonstereotypical Scientists and decreased Stereotypical Scientists).

As the shifts in relatability were comparable for majors and nonmajors across a range of demographics, we combined majors and nonmajors for subsequent analyses. For the Stereotypes prompt, we started deductive analysis using the existing categories from previous coding frameworks: *nonstereotypical descriptors*, *positive stereotypes*, *negative stereotypes*, *fields of science*, *stereotypical scientists*, and *nonstereotypical scientists* (Refs. 8, 9; see Supplemental Table S3). We inductively constructed categories for new emerging data to include *awareness/discussion of inequitable nature of science*, *awareness of health disparitie*s, and *shifts from negative stereotypes to positive/nonstereotypical descriptors*. As shown in Fig. 5, we observed a significant increase in the proportion of students who included nonstereotypical descriptors (e.g., “Anyone can be a scientist”) and a significant decrease in negative stereotypes (e.g., European, elite, white, cis-men) based on McNemar’s χ^2^-analysis (nonstereotypical descriptors: χ^2^ = 82.6, *P* < 2.2e-16; negative stereotypes: χ^2^ = 42.8, *P* = 6.22e-11). Consistent with statistical comparisons in previous studies, the shift in positive stereotypes (e.g., curious, intelligent) was not significant (χ^2^ = 0.338, *P* = 0.561). Notably, as shown in Fig. 6, there was a significant increase in the proportion of students who named nonstereotypical scientists like the names of the scientists featured in the six assignments (χ^2^ = 37.6, *P* = 8.71e-10) and a significant decrease in the names of stereotypical scientists (χ^2^ = 32.6, *P* = 1.14e-8). Please see Table 3 for example quotes illustrating the shift from stereotypical to nonstereotypical scientists. The remaining categories showed low frequencies and/or no significant shifts (see Supplemental Figs. S1–S3).

**Table 3.** Examples of pre- and postresponses to Stereotypes Prompt: “Based on what you know now, describe the types of people that do science. If possible, refer to specific scientists and what they tell you about the types of people that do science”

Pseudonym	Preassessment	Postassessment
Diana	“A lot of scientists that are shown in textbooks are white, cis male and grew up in a stable financial status. I can’t really name specific scientists but a lot of scientists shown in textbooks are also from prestigious schools.”	“I learned that people who do science can do research on specific gender or ethnic groups and not just the standard middle class white population that most studies have. It is important to do specific research on specific populations in terms of preventative health for a specific group. One scientist who did this type of research is Dr. Kiranbir Josan and her research on prevention interventions in high-risk South Asians.”
Sam	“A scientist is an individual that studies the natural world and the interactions between different organisms and environments. The types of people that do science are people who are curious and passionate about the natural world and how things in the natural world work. A specific scientist I know is Rosalind Franklin…”	“Science is a broad subject and can be studied by anyone and used in various ways to help the population. For example, Dr. Day discussed how differences in gene expression result in disparities in cancer between white women and African American women. Through this, methods to resolve disparities can be accounted.”
Ahmad	“I would consider the type of people who do science are PhD’s from different parts of the world. I believe that the body of scientists from around the world is extremely diverse, however, perhaps some scientists have their work overshadowed because of where they are from.”	“Scientists come from many different backgrounds and engage in all kinds of work. Some scientists come from low-income backgrounds, BIPOC communities, and other marginalized groups yet that does not stop them from contributing to the scientific community.”
Emmylou	“The types of people that do science are individuals who are passionate in their curiosities. Scientists like Alexander Fleming or Sir Isaac Newton demonstrate pioneers in the field who stumble upon a finding and pursue the scientific method…”	“The types of people that do science are ordinary people who have a passion for serving their communities. People like Dr. Veronica Ades or Dr. Ben Barres are active advocates for people within their field. They continue to promote awareness and challenge the status quo in science in order for equitable guidelines or healthcare practices.”

BIPOC, Black, Indigenous, and People of Color.

#### Performance/competence, interest, and recognition model misfit.

For the Performance/Competence, Interest, and Recognition (PCIR) instrument, our indices suggested that our data did not fit the model. We lacked empirical evidence for internal structure because the responses did not align with the hypothesized structure of the instrument (37), so here we summarize the sources of the PCIR model misfit. First, from our descriptive statistics, we noticed extensive missing data from the PCIR responses. Even fewer responded to our control question correctly (i.e., “Select ‘Somewhat Disagree’ for this row.”). Despite removing individual responses that contained either missing values or incorrect responses to our control question, we found most factor loadings were below 0.7, suggesting the three factors of “Performance/Competence,” “Interest,” and “Recognition” were not explained by the students’ responses to their associated items. In fact, Horn’s Parallel Analysis (36) for factor retention suggested retaining four factors, providing further evidence that our data did not fit the theoretical model of three factors. Thus, given the lack of evidence for validity for our student population, no statistical comparisons were made on pre- and postresponses from the items of the PCIR instrument.

## DISCUSSION

To diversify the medical field, we must expose undergraduate students who hold minoritized identities to scientific role models who ‘look like them’ (3). In this study, we implemented Scientist Spotlight assignments in one majors and nonmajors physiology course. To assess the impact of these assignments for students from backgrounds that have been excluded from the sciences, we conducted semistructured interviews and used a previously developed pre- and postsurvey. Findings revealed that implementation of these curricular interventions matters, and these assignments increase students’ relatability to scientists and ideas of who can do science. Below, we synthesize key findings from our qualitative and quantitative analyses, draw connections to existing theories in inclusive curricula, and highlight implications for physiology education.

### It is Not Just About Science Content: Humanization of Scientists Resonate with Students

In our interviews, over three-quarters of students resonated with scientists’ biographies, stating the human aspect of scientists as inspiring and meaningful. Students highlighted how the biographies allowed them to view scientists as more than their research, as humans who struggle and hold different identities and backgrounds. This is in stark contrast to how scientists have been historically represented. Cawthron and Rowell (38) highlighted stereotypical depictions of the scientist as, “depersonalized and idealized seeker after truth, painstakingly pushing back the curtains which obscure objective reality, and abstracting order from the flux, an order which is directly revealable to him through a distinctive scientistic method” (p. 32). This paints a narrow view of who can be a scientist: only authoritative experts who bear the ultimate truth about the universe (39, 40). Unsurprisingly, presenting scientific knowledge as detached, fixed, nonnegotiable, and only obtained by ‘geniuses’ may dissuade students from critically questioning the reasons behind scientific beliefs or the rationale for accepting certain scientific ideas (39). According to Hodson (39), this causes intellectual dependency on scientists and can be disempowering for students, especially if their identities are different from those who (stereo)typically do science. Our interview study supports the idea that presenting scientists as they are, fallible humans who struggle, hold identities that have excluded them from science, and share stories about overcoming barriers, can personalize science in inspiring ways.

Moreover, half of our student population shared how learning about each scientist's unique journey to science illuminated their research in a different light. This supports Ahn and colleagues’ (40) work that suggested incorporating biographies into scientists’ discoveries bridge the gap between students’ perception of the scientists and their research. Further, many resonated with the reflection questions of the Scientist Spotlight assignments that assisted them in critically thinking about the types of people who do science. We can further personalize science by combining content-based instruction with counterstereotypical scientists’ biographies and reflective questions. Incorporating the human aspect of science through biographies, reflection questions, and research holds promise, as science instruction has historically been decoupled from counterstereotypical scientists and social factors. Thus changing course from teaching a set of scientific facts to making science and scientists come alive through their personal stories, struggles, triumphs, and research may be one way to reduce STEM attrition rates and diversify representation in science fields.

### It Is Not Just Plug and Play: Implementation Matters

Beyond the usefulness of the structure of Scientist Spotlights for humanizing scientists, students also shared that how the instructor introduced the assignments, assigned grades for credit, and followed-up in class about student responses made a meaningful difference. The clarion call that “implementation matters” will not surprise the countless discipline-based education research (DBER) and practitioners who have shown this in their own work (41). Given that half of the students in our study described aspects of instructor implementation, unprompted, we can interpret students’ insights as critical components that can support fidelity of implementation of inclusive STEM curricula like Scientist Spotlights. Fidelity of implementation “represents the extent to which the *critical components* of an intended educational program, curriculum, or instructional practice are present when that program, curriculum, or practice is enacted” [italics added for emphasis] (31). Critical components are both *structural* and *instructional*. Below, we discuss the structural and instructional critical components of how this inclusive curriculum was implemented to support the fidelity of implementation for future studies using Scientist Spotlights.

The structural critical components ensure the curriculum was assigned as designed (31). For the present study, the instructor included key aspects of the assignment as described by Schinske and colleagues (8). Each Scientist Spotlight from the online database (www.scientistspotligts.org) includes a photo, a brief description of the counterstereotypical scientist, a resource about their research, and a biographical resource. The assignment requires students to review these resources and respond to metacognitive reflective questions, which invite students to consider how these resources connect to course content and/or challenge stereotypes about the types of people who do science. In our interviews, students mentioned at least one and sometimes multiple aspects of the assignment that resonated with them, which confirms that students experienced these structural critical components. Given these findings, instructors who consider removing these critical components, perhaps to shorten the assignment to fit into their course, are cautioned to reconsider.

Meanwhile, instructional critical components factor in the behaviors of instructors and students (31). In our study, students described aspects of the instructor behaviors that stood out to them in relation to the implementation of Scientist Spotlights. These behaviors included how the instructor established an inclusive classroom environment as well as how they introduced and followed up after the homework assignments. The specifics of these instructional choices aligned with what the senior author observed as a coinstructor with the professor who invented and assessed Scientist Spotlights (8, 10). The senior author modeled positively phrased noncontent language (42, 43), promoting inclusion and preframing the assignment along with the practice of grading the assignments (i.e., not just offering extra credit). Below we highlight the aspects related to instructor implementation that students identified in our study to inform evidence-based professional development and fidelity of implementation for future studies on inclusive STEM curricula at large.

First, students emphasized how the classroom environment was conducive to their engagement with an inclusive STEM curriculum like Scientist Spotlights. When the instructor was “insistent on the fact that all of us are capable” (Jocelyn), students recalled and associated this positively phrased noncontent language (42, 43) with their perceptions of the assignment. One may wonder how students’ perceptions of instructor beliefs and values align with the impact of an inclusive STEM curriculum on student outcomes. To explore the impacts of instructors, subsequent studies could bridge research on faculty beliefs with student outcomes before and after the implementation of an inclusive STEM curriculum like Scientist Spotlights.

Second, students shared how the instructor introduced and evaluated the assignment. One student recalled that the instructor said, “just go into these assignments with an open mind and open heart and just learn what these scientists do” (Reina). A couple of students noted how the assignments were graded for course points affected how they engaged with the assignment. Previous studies on Scientist Spotlights at the undergraduate level focused on the assessment and lacked detail on how the assignment was introduced or graded in these wide-ranging contexts (8, 27–30). Our study revealed that instructors’ introduction and follow-up to these assignments could affect student outcomes. Specifically, introducing the scientist during the week in which their research aligns with the course content improves students’ buy-in for why they are learning about these scientists. This finding is similar to another study in which high school teachers who reported engaging their students in a class discussion about Scientist Spotlights had significant shifts in multiple measures of their students’ science identity (9).

Third, students in our study appreciated the weekly in-class follow-up discussion in which the instructor chose a few examples of anonymous reflections from the Spotlight assignments to showcase the diversity of students’ responses. This not only provides evidence for students that the instructor reads and values their responses but also guides students in how to create meaningful reflections.

Even though we did not set out to study instructor implementation, our findings indicate the importance of fidelity of implementation for Scientist Spotlight assignments. Thus future work in developing an inclusive STEM curriculum would benefit from sharing and/or reproducing the structural and instructional critical components outlined here.

### It Is Not Just Memorization: Students Report That Inclusive Curricula Supports Learning and Retention of Physiology Content

In our investigations, students articulated the positive influence of Scientist Spotlight assignments in facilitating their retention of physiology content. To the best of our knowledge, this is the first study that has provided direct evidence of this connection from the voices of undergraduate students. The creator of Scientist Spotlight assignments, Jeff Schinske, quantitatively evaluated the impact of Scientist Spotlights on undergraduate students’ grades in two offerings of the same course: one treatment group who completed these assignments and a control group who completed an alternative assignment (8). Students who completed the Scientist Spotlight assignments achieved higher course grades than the control group. K-12 literature examining the effects of inclusive curricula on students without disabilities also shows a positive or neutral correlation between academic performance and inclusion [see Kart and Kart (44) for a review of this literature]. Other K-12 studies have examined if inclusive environments affect students with disabilities’ performance, with the majority reporting most students with disabilities in high inclusive settings academically outperform those in less inclusive environments (45). These quantitative measures of inclusive curricula and student performance complement our qualitative findings of students’ reflecting on how curricula that highlights counterstereotypical scientists make course content more memorable for students, and thus, may improve course grades (although we did not measure this in our study). More investigations are warranted to confirm if these correlations exist on a broader scale.

Tangential findings by Tripp and colleagues (46) elucidated how anatomy and physiology (A&P) prerequisites impacted the retention of information and perceptions of relevance for nursing professionals. Nurses reported the overabundance of A&P curricular content and details without connection to real life decreased retention of knowledge and was irrelevant to professional nursing careers. Additionally, the excessive focus on A&P content could act as a barrier, excluding otherwise capable individuals from entering the prenursing pathway (46). Our study builds on this literature suggesting that inclusive curricula centering counterstereotypical scientists may be one way to increase students’ retention of knowledge in physiology classrooms by making content relevant and memorable.

### It Is Not Just STEM Majors: Survey Shows How Non-Majors Can Shift in Possible Selves and Scientist Stereotypes Following the Scientist Spotlight Intervention As Well

Following the Scientist Spotlights intervention in majors and nonmajors physiology courses, students significantly shifted from Disagree to Agree with the Relatability prompt, “I know of one or more important scientist(s) to whom I can personally relate.” Interestingly, the proportion of nonmajors who agreed with the prompt nearly quadrupled from 25% who agreed in the presurvey to 80% in the postsurvey. The proportion of majors who agreed with the prompt nearly doubled following the Scientist Spotlights intervention, from 47% initially agreeing with the prompt in the presurvey up to 94% in the postsurvey. These findings show how regardless of students’ intentions or starting points, simply *1*) learning about counterstereotypical scientists in relation to course content, and *2*) reflecting on how such assignments align with or challenge preconceived notions/stereotypes can drive shifts in students’ relatability to scientists. As physiology courses continue to consider inclusive practices to increase student engagement and retention (47–51), the present study shows how Scientist Spotlight assignments, implemented as designed and described here, have strong evidence to advance these efforts.

### Limitations and Future Directions

The present study investigated student perceptions of inclusive physiology curricula through Scientist Spotlight assignments at an emerging Hispanic-serving R1 university on the West Coast. This was an ideal launch point to explore how and which aspects of these curricular supplements impact undergraduate students. However, this sample is limited to a specific region and demographic milieu that is not representative of other institution types (e.g., Community Colleges, Primarily Undergraduate Institutions, Historically Black Colleges and Universities). Extended efforts across these various landscapes are warranted to draw parallels on the most salient portions of Scientist Spotlight assignments. Importantly, Scientist Spotlights were initially designed, implemented, and assessed in a community college setting (8). Given how well Scientist Spotlight assignments align with the guidelines for universal design (52), providing multiple means of engagement, representation, and action and expression for physiology courses, future research can explore how student perceptions of these assignments correspond to learning outcomes for students from a range of backgrounds.

Another limitation of the present study is that the students were recruited from courses taught by the senior author. Performing a study on courses in which one of the authors is the instructor may introduce social desirability bias (53). Students may have felt unable to fully share their perspectives knowing their instructor was also the lead on this project. We made efforts to mitigate this by having an undergraduate researcher perform the interviews and de-identify interview responses, as well as an outside researcher collect and analyze anonymous survey data. We would encourage future studies that assess the impact of Scientist Spotlights in their courses to minimize the impact of these biases through collaborative efforts with undergraduate researchers.

## DATA AVAILABILITY

Data will be made available upon reasonable request.

## SUPPLEMENTAL MATERIAL

10.6084/m9.figshare.26305123 Supplemental Materials *A–D*, Tables S1–S3, and Figs. S1–S3 are available at: https://doi.org/10.6084/m9.figshare.26305123.

## DISCLOSURES

No conflicts of interest, financial or otherwise, are declared by the authors.

## AUTHOR CONTRIBUTIONS

D.O., A.R.A.-P., A.A., J.F.G., D.T., and B.T. conceived and designed research; D.O., A.R.A.-P., A.A., J.F.G., D.T., and B.T. performed experiments; D.O., A.R.A.-P., A.A., J.F.G., D.T., and B.T. analyzed data; D.O., A.R.A.-P., A.A., J.F.G., D.T., and B.T. interpreted results of experiments; D.O., A.R.A.-P., and B.T. prepared figures; D.O., A.R.A.-P., and B.T. drafted manuscript; D.O., A.R., and B.T. edited and revised manuscript; D.O., A.R.A.-P., A.A., J.F.G., D.T., and B.T. approved final version of manuscript.
